# Supplementary material for: Co-Stimulatory and Immune Checkpoint Molecule Expression on Peripheral Immune Cells Differs Age Dependently Between Healthy Donors and Patients with Head and Neck Squamous Cell Carcinoma
Source: Cancers (Basel). 2025 Oct 2;17(19):3215. doi: 10.3390/cancers17193215 (PMC12523874; doi:10.3390/cancers17193215)
Supplement: Supplementary file 1 [file cancers-17-03215-s001.zip › cancers-3862457-supplementary.pdf]

Table S1. Clinicopathological features of healthy donors.

Clinicopathological features of the healthy donors	
**Number**	**Diagnosis**
14	None
4	Tonsillectomy
2	OSAS
1	Deviated nasal septum
1	Chronic sinusitis
1	Vocal chord polyp
1	Adenoma Gl. parotidea
1	Chronic laryngitis
1	Hypopharyngeal diverticulosis
1	Spasmodic dysphonia
1	Lateral neck cyst
1	Chronic inflammation of Gl. submandibularis
1	Cholesteatoma

Table S2. Staining panels and detailed antibody information.

Checkpoint Panel 1							
**Channel**	FL1	FL2	FL4	FL5	FL7	FL8	FL9
**Color**	FITC	PE	PC5	PE-Cy7	AF700	APC-Fire750	BV 421
**Antibody**	CD8	PD1	CTLA4	CD39	CD4	CD19	BTLA

Checkpoint Panel 2							
**Channel**	FL1	FL2	FL4	FL5	FL7	FL8	FL9
**Color**	FITC	PE	PC5	PE-Cy7	AF700	APC-Fire750	BV 421
**Antibody**	CD8	CD137	CD27	CD39	CD4	CD19	GITR

Checkpoint Panel 3							
**Channel**	FL1	FL2	FL4	FL5	FL7	FL8	FL9
**Color**	FITC	PE	PC5	PE-Cy7	AF700	APC-Fire750	PB
**Antibody**	CD8	LAG3	OX40	CD39	CD4	CD19	TIM3

Name / Color	Clone	Specimen	Order number	Company
BTLA Brilliant Violet 421™	MIH26	human	344512	BioLegend
CD137 PE	4B4	human	12-1379-42	eBioscience
CD19 APC-Fire 750	HIB19	human	302257	BioLegend
CD27 Pe-Cy5	0323	human	15-0279-42	eBioscience
CD39 PE-Cy7	eBioA1	human	25-0399-42	eBioscience
CD4 Alexa Fluor 700	OKT4	human	56-0048-82	eBioscience
CD45 AMCyan	2D1	human	339192	BD Bioscience
CD8 FITC	SK1	human	9011-0087-120	eBioscience DX
CTLA4 PE-Cy5	BNI3	human	561717	BD Bioscience
GITR Brilliant Violet 421™	108-17	human	371207	BioLegend
LAG3 PE	3DS223H	human	12-2239-42	eBioscience
OX40 PE-Cy5	Ber-ACT35	human	350009	BioLegend
PD-1 PE	eBioJ105	human	12-2799-41	eBioscience
PD-L1 PE	MIH1	human	12-5983-42	eBioscience
TIM3 PB	F38-2E2	human	345041	BioLegend

Table S3. Summary of all expression analysis results including the respective statistics and trends.

Cell population	Checkpoint molecule	Patient cohort	p-value	Correlation coefficent r (from 1 to -1)	Expression
CD8+ T cells	LAG3	Healthy	0,0282	-0,4007	↓↓
		HNSCC	0,2088	0,2115	↑
	PD1	Healthy	0,0072	-0,4802	↓↓
		HNSCC	0,1981	0,2165	↑
	BTLA4	Healthy	0,0027	-0,5359	↓↓
		HNSCC	0,5902	-0,09149	≈
	CD137	Healthy	0,0470	-0,3719	↓↓
		HNSCC	0,9142	-0,01835	≈
	CD27	Healthy	<0,0001	-0,7377	↓↓↓
		HNSCC	0,0169	-0,3903	↓↓
CD4+ T cells	LAG3	Healthy	0,0349	-0,3865	↓↓
		HNSCC	0,0321	0,3530	↑↑
	PD1	Healthy	0,4550	-0,1417	↓
		HNSCC	0,0005	0,5453	↑↑
	BTLA4	Healthy	0,0230	-0,4209	↓↓
		HNSCC	0,0019	-0,4932	↓↓
	CTLA4	Healthy	0,0084	-0,4803	↓↓
		HNSCC	0,0857	0,2905	↑↑
	GITR	Healthy	0,0404	-0,3763	↓↓
		HNSCC	0,3659	0,1530	↑
	CD27	Healthy	0,0219	-0,4240	↓↓
		HNSCC	0,0018	-0,4970	↓↓
CD4+CD39+ T cells	PD1	Healthy	0,1963	-0,2427	↓
		HNSCC	0,1502	0,2413	↑
	BTLA4	Healthy	0,0010	-0,5794	↓↓
		HNSCC	0,2875	-0,1796	↓
	CTLA4	Healthy	0,0475	-0,3711	↓↓
		HNSCC	0,1546	0,2423	↑
	GITR	Healthy	0,0209	-0,4199	↓↓
		HNSCC	0,8127	0,04032	≈
	CD137	Healthy	0,0183	-0,4353	↓↓
		HNSCC	0,9206	-0,01698	≈
	CD27	Healthy	0,4481	-0,1465	↓
		HNSCC	0,0205	0,0205	≈

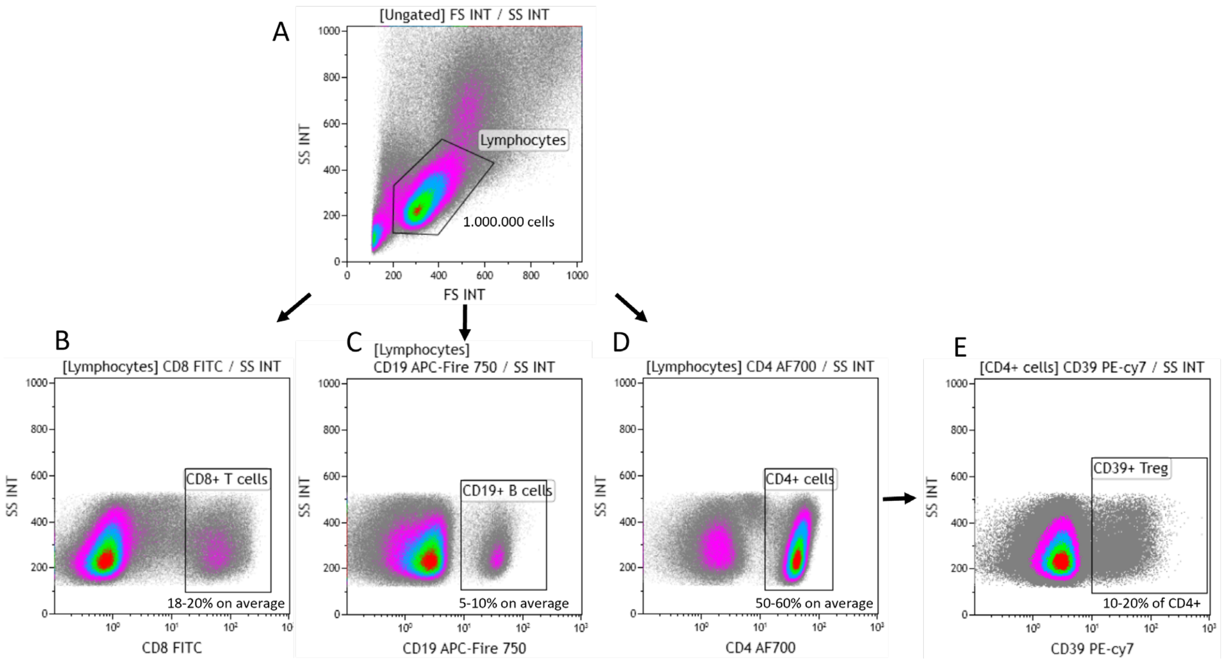

Figure S1. Flow cytometry gating strategy used for selection of the lymphocytes (**A**) the CD8⁺ (**B**), CD4⁺ (**C**) and CD39⁺ (**D**) T cells.

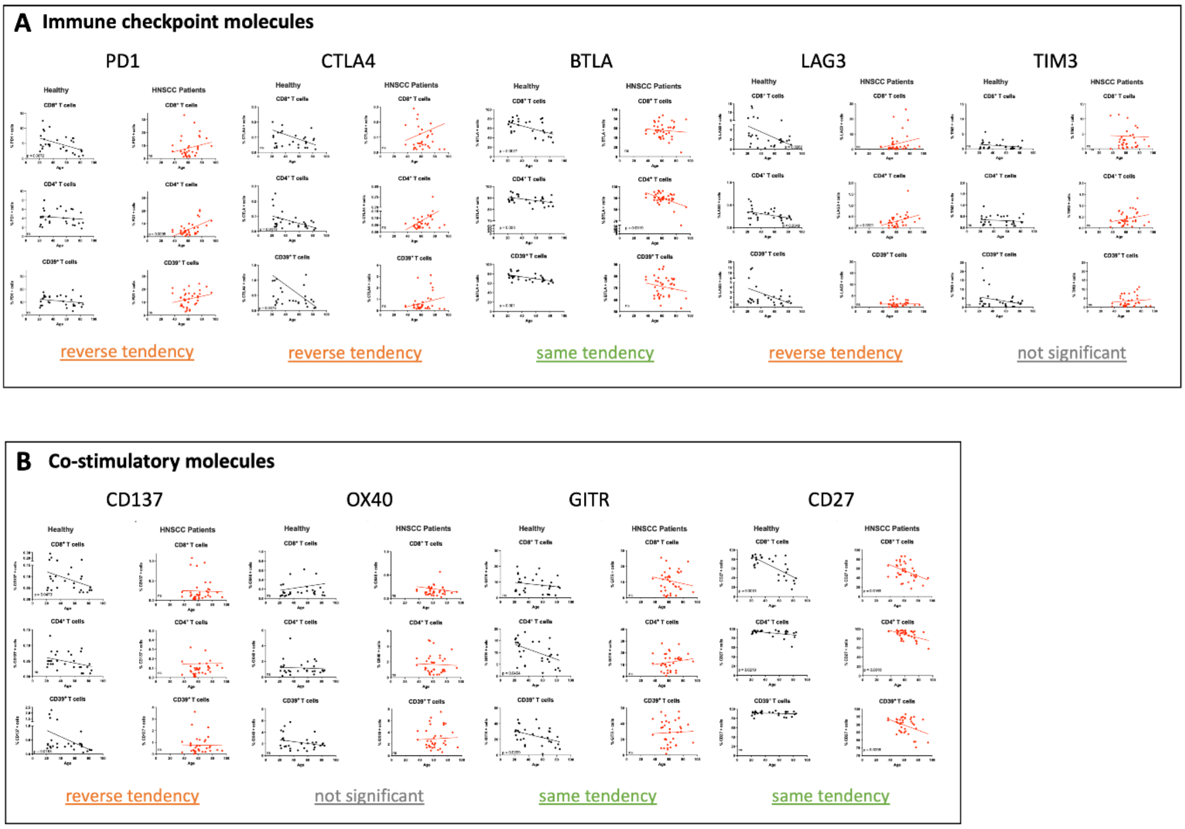

Figure S2. Dot plots depict the expression of immune checkpoint molecules (ICMs) on CD8+, CD4+ and CD39+ T cells for healthy donors (black) and HNSCC samples (red). The straight line represents Pearson's correlation analysis along with the corresponding p-values. **A**: all co-inhibitory ICM **B**: all co-stimulatory molecules. *ns = not significant.*

A

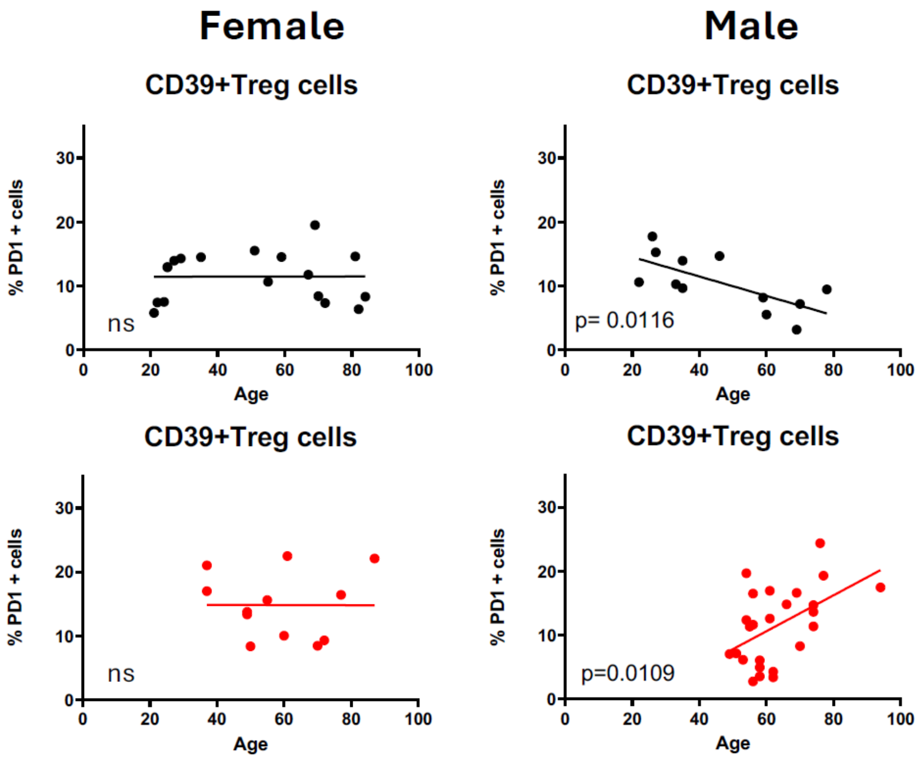

B

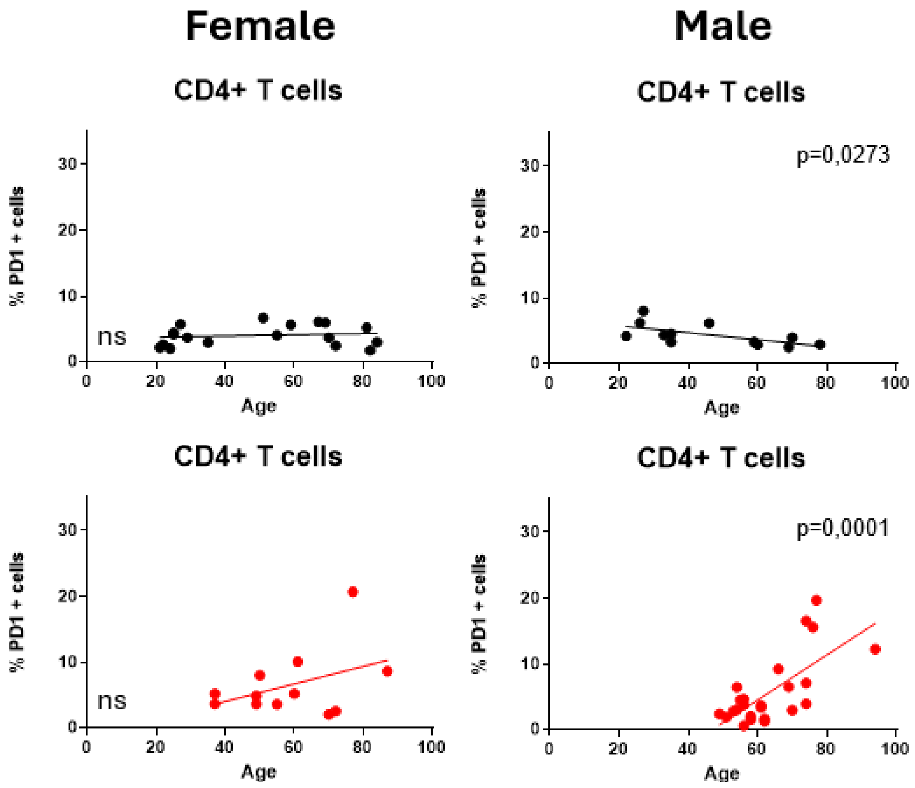

Figure S3. Dot plots depict the expression of immune checkpoint molecules (ICMs) on CD39+ T cells and CD4+ T cells for healthy donors (black) and HNSCC samples (red). The straight line represents Pearson's correlation analysis along with the corresponding p-values. **A**: PD1-Expression on CD39+ Treg cells **B**: PD1 Expression on CD4+ T cells. *ns = not significant*.
